# Elevated-temperature-induced acceleration of PACT clearing process of mouse brain tissue

**DOI:** 10.1038/srep38848

**Published:** 2017-01-31

**Authors:** Tingting Yu, Yisong Qi, Jingtan Zhu, Jianyi Xu, Hui Gong, Qingming Luo, Dan Zhu

**Affiliations:** 1 Britton Chance Center for Biomedical Photonics, Wuhan National Laboratory for Optoelectronics, Huazhong University of Science and Technology, 1037 Luoyu Road, Wuhan, Hubei 430074, China; 2 Department of Biomedical Engineering, Key Laboratory of Biomedical Photonics, Ministry of Education, Huazhong University of Science and Technology, 1037 Luoyu Road, Wuhan, Hubei 430074, China

## Abstract

Tissue optical clearing technique shows a great potential for neural imaging with high resolution, especially for connectomics in brain. The passive clarity technique (PACT) is a relative simple clearing method based on incubation, which has a great advantage on tissue transparency, fluorescence preservation and immunostaining compatibility for imaging tissue blocks. However, this method suffers from long processing time. Previous studies indicated that increasing temperature can speed up the clearing. In this work, we aim to systematacially and quantitatively study this influence based on PACT with graded increase of temperatures. We investigated the process of optical clearing of brain tissue block at different temperatures, and found that elevated temperature could accelerate the clearing process and also had influence on the fluorescence intensity. By balancing the advantages with drawbacks, we conclude that 42–47 °C is an alternative temperature range for PACT, which can not only produce faster clearing process, but also retain the original advantages of PACT by preserving endogenous fluorescence well, achieving fine morphology maintenance and immunostaining compatibility.

High-resolution mapping of neuronal connectivity through the entire brain is indispensable for understanding how the brain functions. Due to the high turbidity of biological tissues, it is necessary to make thin sections for the widespread histological approaches, which are labor-intensive and time-consuming. The automated serial-sectioning and imaging techniques have been developed recently to obtain high-resolution atlas of the mouse brain with high-throughput^1^,^2^,^3^. The major challenges of these sectioning methods involve tissue deformation induced by mechanical slicing, efficient processing of massive data sets and destruction of the samples^4^.

Tissue optical clearing has emerged as a distinct approach for imaging deeper in large volumes by reducing the scattering and improving the light penetration depth with kinds of optical clearing agents or tools^5^,^6^,^7^,^8^,^9^,^10^,^11^,^12^. The rise of these techniques shows a great potential for obtaining the three-dimensional high-resolution images of un-sectioned whole brain by combining with various optical imaging techniques^13^,^14^,^15^, which provides a new perspective for visualization of brain-wide neuronal networks. Recently, to achieve transparency, different methods have been developed. As an organic solvent-based optical clearing method, 3DISCO could make the brain and spinal cord resemble glass with limited storage time owing to fluorescence quenching^16^,^17^,^18^. Some other water-based approaches were developed to achieve better fluorescence preservation. Sca*l*e is based on urea to clear the whole mouse brain but limited on tissue fragility and long incubation time^19^. Sca*l*eS is a further development of Sca*l*e based on sorbitol and urea with structural stability and shorter time^20^. *Clear*^*T2*^ takes a short time to clear the whole embryos based on formamide and polyethylene glycol but shows less effect on adult brain^21^. SeeDB and SeeDB2 are methods based on fructose or iohexol without tissue deformation^22^,^23^,^24^,^25^,^26^ and showed modest clearing capability. The subsequent aqueous methods like CUBIC^27^,^28^ not only clear the whole brain with good transparency and fluorescence preservation, but also can achieve large-volume tissue immunostaining.

CLARITY, as a fundamentally distinct approach, transformed the brain tissue into hydrogel-tissue hybrid and introduced electrophoretic tissue clearing for lipid extraction to achieve high transparency^29^,^30^,^31^,^32^,^33^,^34^. However, this method could be challenging to implement for researchers and get unstable tissue quality. This led to the development of protocols that render tissue transparent through passive delipidation techniques, including advanced CLARITY without electrophoretic clearing^31^, PACT^32^. The procedure for PACT is similar to CLARITY method, including the hydrogel-tissue hybridization to provide a physically tissue support framework, passive lipid extraction with ionic detergents and embedding in high refractive index agent for imaging. However, as one of the passive clearing methods, PACT is characterized by inherently slow speed like TDE-based clearing method^4^,^35^,^36^,^37^,^38^ and FRUIT^39^. Generally, the optical clearing methods process the samples at room temperature or 37 °C. It has been mentioned that higher temperature could be used for faster clearing, such as 50 °C in SeeDB37ht^22^ or 60 °C in passive CLARITY^31^. They both indicated that high temperature might cause partial quenching of fluorescent proteins, but lack of quantitative analysis^22^,^31^. The detailed investigation on the influence of high temperatures on passive optical clearing is in absence. It is also critical to balance the clearing speed and fluorescence preservation.

In this work, based on the original PACT, we aim to systematically study the influence of high temperature on the clearing and seek to screen the alternative temperature range, which can not only enhance the clearing efficiency, but also preserve the fluorescence of protein whose stability is sensitive to temperature. The process of optical clearing of brain tissue block at different temperatures (37–52 °C) and the influence on the fluorescence intensity were examined by bright-field images and the fluorescence images, respectively. In addition, the effect of elevated-temperature on morphology preservation of samples and the compatibility with immunostaining for tissue block were investigated.

## Results

High temperature can promote the random molecular motion, which can accelerate the diffusion of the chemical reagents into biological tissues. Generally, room temperature and 37 °C are chosen as the processing temperature in most of optical clearing methods due to the thermal sensitivity of fluorescent proteins, such as GFP^40^, which leads to longer incubation time. To achieve rapid tissue clearing, we quantitatively evaluated the optical transparency and fluorescence intensity of mouse brain blocks incubated at 37 °C, 42 °C, 47 °C, 52 °C, 57 °C, respectively, and balanced with these two parameters to determine the alternative temperatures for PACT. Meanwhile, we investigated the increase of imaging depth, the tissue morphologic maintenance, and immunostaining compatibility for brain blocks at indicated temperatures.

### Optical transparency with different temperatures

**Figure 1:**
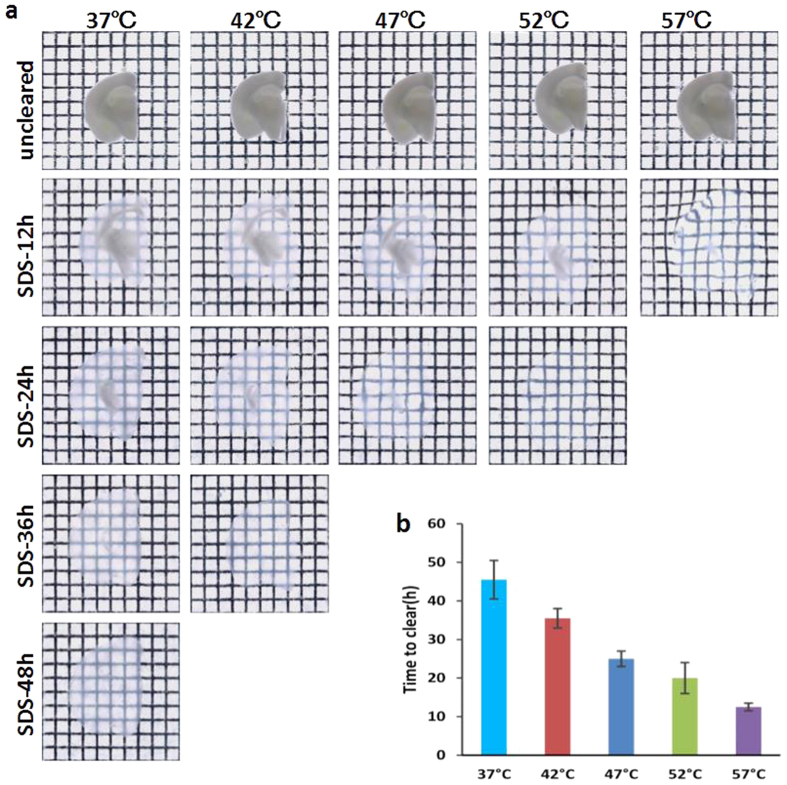
Optical transparency with different temperatures. (**a)** Bright-field images of 1-mm-thick mouse coronal blocks at different temperatures in SDS solution with time. Grid size, 1.45 mm × 1.45 mm. “uncleared” indicates the sample with only hydrogel-embedded and placed in PBS. “SDS-12hr”, “SDS-24hr”, “SDS-36hr”, and “SDS-48hr” indicate 12-, 24-, 36-, 48-hours SDS clearing. (**b**) A comparison of time to clear for different temperatures.

The optical transparency of the 1-mm-thick mouse brain coronal block was observed after 0-, 12-, 24-, 36- and 48-hours SDS (Sodium-Dodecyl Sulfate) clearing. The representative images of the SDS-cleared brain slices through the time course photographed in the trans-illumination mode are shown in Fig. 1a. The time to clear for brain slices in 8% SDS solution at 37 °C, 42 °C, 47 °C, 52 °C, 57 °C is respectively recorded and compared, as showed in Fig. 1b. The results demonstrate that with the increase of temperature, the time to clear reduced and the clearing speed is increased.

After clearing in SDS for 6 hours, the transparency of samples appears different among five temperatures (Supplementary Fig. 1a). Hence, to quantify tissue transparency, 6-hours was taken to compare the collimated transmittance of five temperatures with SDS-clearing and further sorbitol incubation for refractive index matching. The relative transmittance at the wavelength of 500 nm for 37 °C, 42 °C, 47 °C, 52 °C, 57 °C are 5.5%, 10.4%, 18.9%, 30.3%, 57.9%, respectively. Then incubated in sorbitol solution, the transmittance at 500 nm increase to 32.5%, 45.2%, 58.6%, 69.2%, 86.5% for five temperatures, respectively. This refractive index matching process with sorbitol after PACT could increase the transmittance of the brain samples (Supplementary Fig. 1b). For SDS and SDS-sorbitol clearing samples, the transmittance values both increase with the increase of temperature.

It is concluded that processing with higher temperature could achieve better transparency with higher transmittance at the same incubation time and accelerate the clearing process.

### Fluorescence preservation of neurons in different temperatures

**Figure 2:**
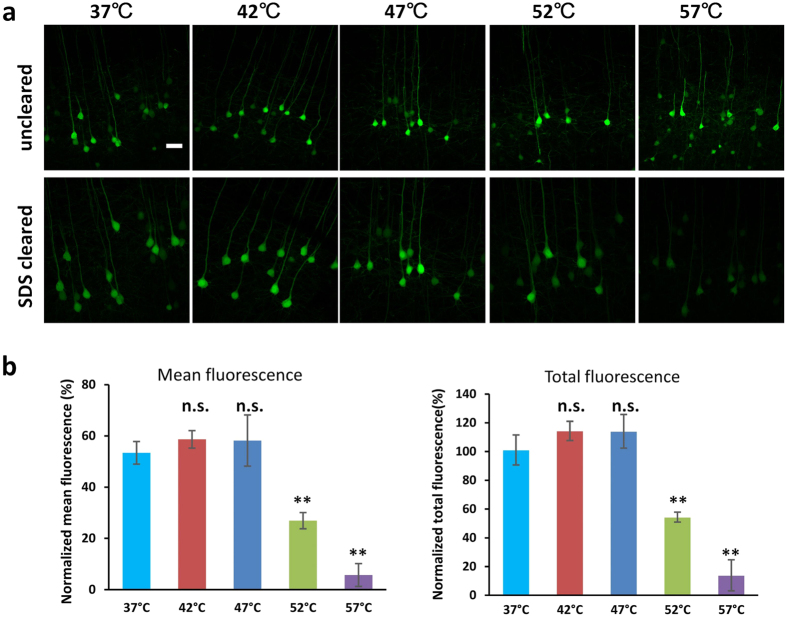
GFP Fluorescence intensity of neurons before and after SDS clearing in gradient temperature. (**a**) Each image is a 50-μm maximum projection of image stacks acquired with confocal microscope (10×/0.5 objective). Scale bar, 50 μm. (**b**) Quantitative calculation of relative mean fluorescence intensity and total fluorescence intensity. Error bars denote standard deviations. (n.s. = not significant, ∗∗P < 0.01).

Considering proteins are sensitive to temperature and SDS, we imaged the neurons of mouse brain cortex before and after clearing with SDS in different temperatures, then examined the fluorescence intensity of GFP under identical conditions. The results from the 50-μm maximum projections of z-stack images show that higher temperature (52 °C and 57 °C) can induce obvious decrease in GFP fluorescence intensity (Fig. 2a). Then, the mean fluorescence intensity of SDS cleared 1-mm-thick brain slices were calculated. We found that there was a decrease in mean fluorescence intensity after cleared under all temperatures (37–57 °C). Taking tissue expansion into account, we observed that the total fluorescence intensity increased to 1.18 ± 0.11 for 42 °C and 1.08 ± 0.17 for 47 °C, which were comparable to 37 °C, while present highly significant reduction for 52 °C and 57 °C. Considering the clearing effect described above with the preservation of fluorescent signals, the results demonstrate that 42–47 °C give it an ideal compromise between clearing speed and fluorescence preservation. Further, the relative fluorescence intensity along with time for different temperatures was shown in Supplementary Fig. 2. The statistical analyses show that there is no difference in fluorescence change within 48 hours between 42 °C and 37 °C, but extremely significant between other higher temperatures (47–57 °C) and 37 °C.

### Imaging depth of mouse brain blocks with different temperatures

**Figure 3:**
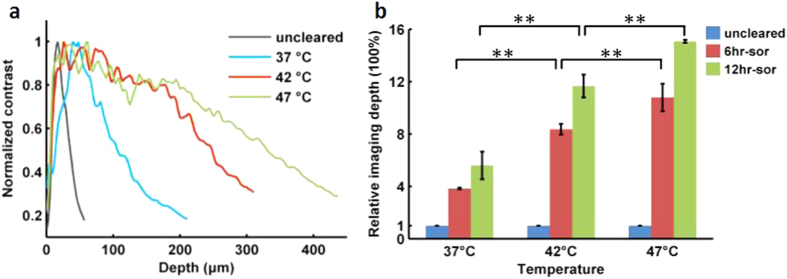
Quantification of imaging depth with different temperatures. (**a**) Image contrast for 1-mm-thick *Cx3Cr1*-GFP brain sections before and after 6-hours SDS clearing followed by sorbitol incubation. The imaging depth is determined where the contrast drop to 1/e from the surface. (**b**) Increase of imaging depth with clearing time for 37 °C, 42 °C and 47 °C. Error bars denote standard deviation. “6hr-sor” and “12hr-sor” indicate 6-hours and 12-hours SDS clearing followed by sorbitol incubation, respectively (∗∗P < 0.01).

The imaging depth is another important parameter concerned in optical clearing methods for fluorescence imaging techniques except for the transparency and fluorescence preservation. In this study, the imaging depth for different temperatures was also quantified based on contrast attenuation. To understand the correlation of time-to-clear and imaging depth, the fluorescence of *Cx3Cr1*-GFP brain samples after clearing with SDS at typical time points (6 hours and 12 hours) followed by sorbitol immersion were acquired to calculate the imaging depth, as shown in Fig. 3. Figure 3a gives the typical contrast attenuation of 1-mm-thick brain sections before and after 6-hours SDS clearing followed by sorbitol incubation, and the imaging depth is determined at the point where the contrast reduced to 1/e of the maximum value on the surface. As shown in Fig. 3b, the imaging depth increases with SDS-clearing time for all three temperatures, and 47 °C shows larger increase of imaging depth than 42 °C at two time points. When applied to 3-mm-thick brain blocks, the results are similar (Supplementary Fig. 3).

### Tissue size and morphologic change during the clearing process

**Figure 4:**
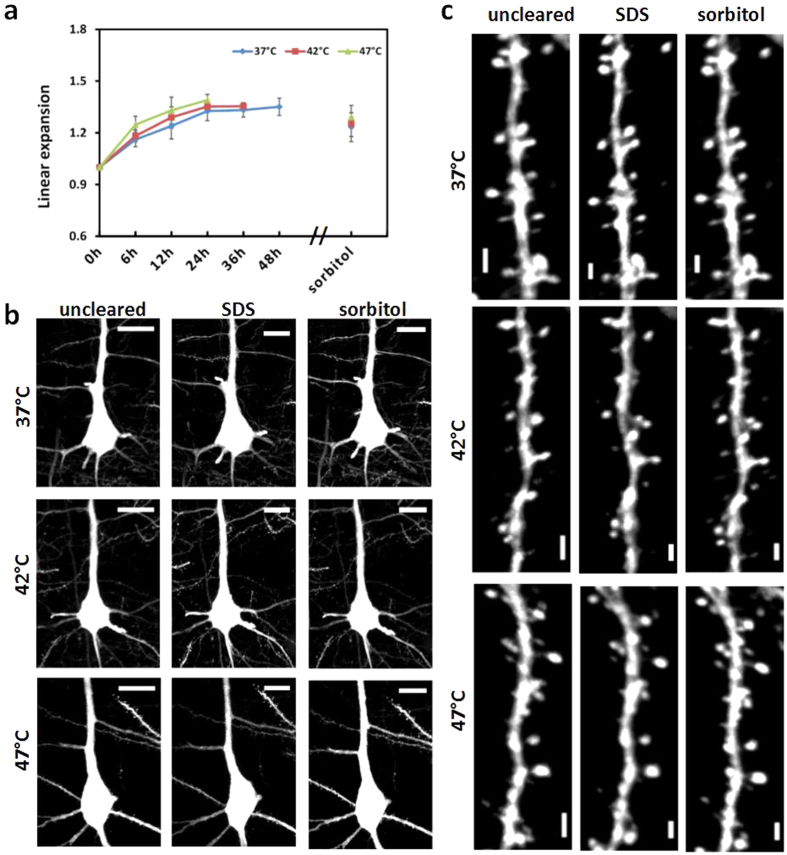
Tissue size change and neuronal morphology. (**a**) Sample expansion through SDS clearing at different temperatures and sorbitol incubation. Error bars denote standard deviation. (**b**) Typical neurons of the *Thy1*-GFP-M mouse cortex before and during clearing. The images were acquired with confocal microscope (20×/0.8 dry objective). Scale bar, 20 μm. (**c**) Structure of dendrite spines before and during clearing process. Scale bar, 2 μm. Each image is a maximum projection of image stacks. “uncleared” indicates the sample with only hydrogel-embedded. “SDS” indicates the sample cleared with SDS. “Sorbitol” indicates the sample with further sorbitol incubation after SDS clearing.

Tissue morphology preservation is of particular importance to analyze fine structures of biological tissues. During the clearing process, the changes of sample size are inevitable due to the dehydration or hydration of different agents. The linear expansion during 37 °C, 42 °C, and 47 °C-SDS clearing and sorbitol incubation were calculated, as shown in Fig. 4a. After sorbitol incubation, the tissue expansion is diminished and the size of slices reduces to similar values for three temperatures. To characterize the effects on neuronal morphology, typical neurons of the *Thy1*-GFP-M mouse cortex were imaged before clearing and during clearing process. The main structures of the neurons were preserved well, as shown in Fig. 4b. After cleared with SDS, the locations of a few dendrites show slight shifts. Then with sorbitol incubation, the shift decreases and the dendrites present close to original location. This deformation is anticipated to be related to the tissue expansion. Figure 4c show the images of dendrite spines before and during the clearing process. The results indicate that the fine structures were all preserved well for 37 °C, 42 °C, and 47 °C.

### Compatibility with immunostaining of brain block after PACT

**Figure 5:**
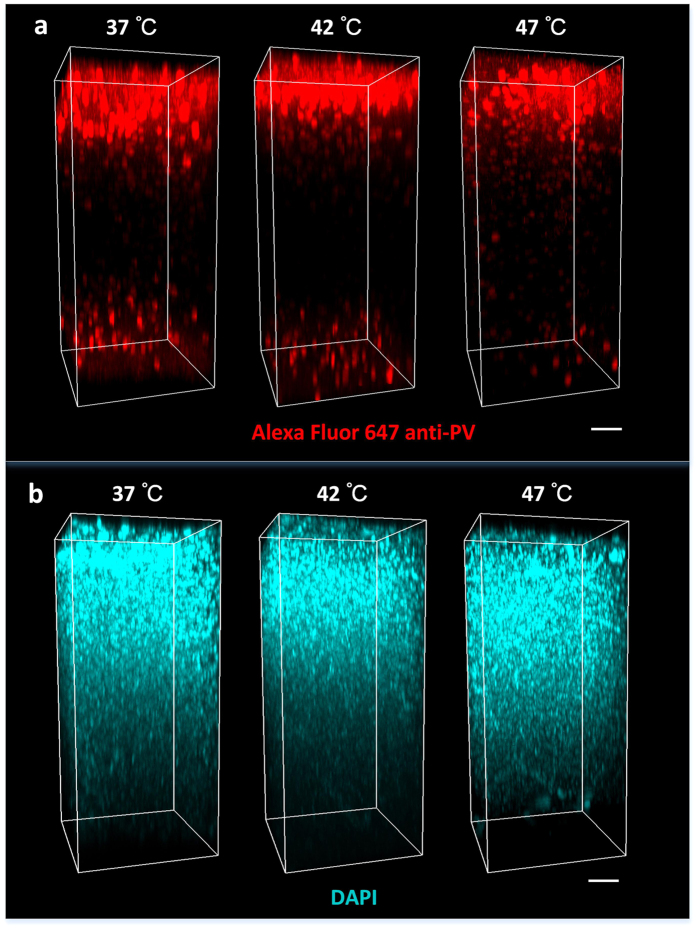
Immunostaining of 1-mm-thick brain slices through PACT with different temperatures. Cleared brain slices with PACT in different temperatures were immunostained for (**a**) parvalbumin (Alexa Fluor 647 anti-PV) and (**b**) nuclei stained with DAPI, respectively, then incubated in sorbitol solution for 5 h and mounted for imaging. Scale bar, 100 µm.

To investigate whether the raising temperature during PACT clearing impedes the subsequent immunostaining, we used anti-parvalbumin (PV) antibody to immunostain the cleared 1-mm-thick mouse brain sections with PACT in different temperatures, and focused on the success of labeling. The effectiveness of immunolabeling (after SDS incubation at 47 °C) was proved by staining the GFP protein, which is an endogenous protein that can be used as comparative reference for complete labeling (Supplementary Fig. 4). Further, Fig. 5 demonstrates the immunostaining anti-parvalbumin signal (Alexa Fluor 647) and the nuclei staining with DAPI. The imaging parameters used for these images are identical. As we can see from Fig. 5, the immunostaining and nuclear staining after PACT under all the three temperatures (37 °C, 42 °C, 47 °C) show successful antibody labeling at cleared state with respective clearing time. These results indicate that moderately rising temperature for PACT works well with subsequent immunostaining.

## Discussion

As mentioned above, there’re a number of well-established optical clearing methods. Thereinto, PACT is an aqueous-based passive clarity technique suitable for passive lipid extraction of 1- to 3-mm-thick tissues^32^. Previous studies mentioned that the use of higher temperature could quicken the clearing process and claimed that it might quench the fluorescent signal without detailed evaluation^22^,^31^. As is well-known, most proteins are sensitive to temperature, for instance, the stability of GFP could be reduced with the increase of temperature^41^. In this work, with systematic investigation from clearing speed, fluorescence preservation, and morphology maintenance to immunostaining compatibility, an appropriate temperature range has been determined as the alternative choice for the researchers instead of 37 °C in original PACT. It has certain scope of application, especially for the passive clearing methods need long incubation time, an appropriate increased temperature is conducive to save time and accelerate the clearing process under such circumstances. It is noticed that the thermal acceleration is unapparent for those solvent-based methods^15^,^16^,^17^,^18^ and active clearing methods^29^,^30^.

During the PACT clearing procedure, we found that the brain slices treated at 57 °C are too soft and fragile to handle for imaging easily, which indicates that higher temperature is not beneficial to tissue morphology maintaining. For the other four temperatures, the clearing protocol can also lead to tissue expansion in certain extent, as shown in Fig. 1a. In the investigation of fluorescence preservation, the field of view of images for uncleared samples in Fig. 2a is slightly larger than the cleared state owing to the tissue expansion of SDS clearing, which is common to the other detergent-clearing methods. Tissue expansion is most likely to induce structure rupture or deformation, such as Sca*l*eA2^20^, while some clearing methods which do not cause obvious change in tissue volume can achieve fine morphology preservation, such as SeeDB^22^,^23^,^24^,^25^. This expansion and deformation could be diminished by introduction of refractive index matching solution, such as sorbitol^32^, fructose^22^,^23^,^24^,^25^ and histodenz^26^,^31^. Considering the cost and viscosity of the chemical agents, sorbitol was employed to achieve the refractive index matching in this study. What is noteworthy is that the samples need sufficient rinsing to remove the SDS before incubating in sorbitol. Otherwise, the samples would turn white in a few hours and the tissue transparency would decrease, which made imaging deeply difficult.

Though the higher temperature can accelerate the clearing speed, as described above, but is unfavorable for preserving fluorescence of protein which is sensitive to temperature, like GFP^40^. Hence, it is necessary to determine an appropriate temperature range to balance the both. In this study, the GFP fluorescence intensity was quantified based on the fluorescence images, while GFP protein loss was not measured. During detergent-based clearing process, GFP protein is easy to be eluted from tissues, this might be more serious at higher temperatures because the faster clearing rate means it is easier to overclear tissues. In the future work, the protein loss for each temperature should be measured with NanoDrop, as described in the original literature^32^,^33^.

In the immunostaining of brain blocks, the intensity of immunostaining signal in the middle volume was lower than that in the superficial volume, especially for the neuronal fibers. During the passive permeating process of primary and secondary antibodies, it is inevitable for the macromolecules to form concentration gradient from edge to center of samples, which would reflect in the difference of fluorescence intensity. 37 °C is an effective temperature for PACT to immunostain the 1-mm-thick slices^32^,^33^. In this study, 42–47 °C is suggested as the processing temperature in PACT based on the measurements of transparency, fluorescence preservation and imaging depth. The compatibility with immunostaining is also an important factor that should be considered in the clearing method. To address this doubt, the immunostaining for GFP after 47 °C-SDS clearing was carried out to prove the effectiveness of labeling. Further, the immunostaining for parvalbumin and nuclear staining after SDS clearing at 37 °C, 42 °C, and 47 °C were demonstrated to investigate the labeling ability of the antibody and small molecules. The results show that high temperature is compatible to the immunostaining, but cannot aid the antibody penetration due to the same hydrogel formulation (A4P0), which decides the crosslink density of tissue-hydrogel hybrid and pore size that directly affects the diffusion rate.

It should be noted that the immunofluorescence is not suitable for quantitative analysis and comparison due to the variety of interference factors, including individual differences of samples and the important anthropic factor in the complicated operations. And the repeated trials also showed different intensity level (Fig. 5 and Supplementary Fig. 5). This is due to the difficulty of keeping only one variable in all the experiments, the imaging region, the placement angle of samples and even the contact of brain slices with the sample-containing tubes can all influence the staining effect. Hence, we investigated the compatibility of the increased temperatures with immunostaining by focusing on investigating whether the labeling is successful. Though the results above showed successful labeling for all the three temperatures at indicated clearing time, it is uncertain if the antigenicity was influenced due to lack of quantitative measurement of antigenicity. It is worth to note that the antigenicity must be effected with increased temperature in SDS through a longer time accumulation, even with the 37 °C in original PACT^33^. Theoretically, lower temperature is better for the immunostaining. However, in practical applications, if the protocol can satisfy the imaging requirements with faster process, for example, 37 °C instead of 4 °C in traditional studies, or higher temperature instead of 37 °C in this study, it should be useful and valuable.

Based on the physically and chemically support of hydrogel-tissue hybridization, the lipids and unconjugated biomolecules were removed from hybrid with the ionic detergent (SDS). The concentration of SDS solution is critical for the clearing, as demonstrated in the original paper for PACT^32^,^33^. Temperature is an important factor that affects micellar composition. With the increase of temperature, the volume of micelle decreases for SDS solution and the number of micelle increases. For the tissue-hydrogel matrix with certain pore size, it is supposed to be easier for smaller micelles to diffuse, which might explain why high temperature can speed up the clearing process^33^,^42^. Except for the effect on micelle size, high temperature can also affect the stability of the hybrid mesh and the architecture of other biomolecules, which can be investigated with ultrastructural examination using electron microscopy that we lacked. Instead, we evaluated the influence of temperature on the microstructure, e.g. spine, the fine structure on dendrite, as described above.

Though for 1-mm-thick brain sections, the samples could achieve good transparency with fine fluorescence preservation for both 42 °C and 47 °C, a faster decrease in GFP fluorescence was observed under 47 °C (Supplementary Fig. 2) due to the instability of GFP in SDS solutions under high temperatures^41^. Considering in conjunction with the potential impact on antigenicity of labeling target under high temperature, it is suggested that for relative smaller sample (e.g. 1-mm-thick brain section), 47 °C is suitable for acceleration of clearing, but for thicker samples need longer time to clear, lower temperatures are suggested, such as 42 °C, even lower. As shown in Fig. 2b, 42 °C and 47 °C show comparable fluorescence intensity to 37 °C by comparing the fluorescence intensity of different temperatures at cleared state with different clearing time needed for the brain samples (less time for higher temperature). The comparable mean or total fluorescence of GFP signal at the first period of clearing time for 42 °C and 47 °C is supposed to be induced by the alkalinity of clearing solution (pH = 8.5)^43^,^44^, whose effect on the fluorescent signal counteracts the influence of temperature and SDS. In addition, we also investigated the fluorescence preservation at different temperatures for YFP and found that the fluorescent signal decreases with the elevated temperature (Supplementary Fig. 6). Hence, in this study, we set a range for the alternative temperatures suitable for GFP in PACT but not strictly limited, which can be adjusted according to the requirements in certain conditions, including the sample thickness, the tracer thermal properties, the stability of antigenicity of immunolabeling target and so on.

This work demonstrates the temperature rise could enhance the efficacy of PACT, the demonstration on thicker blocks should be carried out in the future work. It is anticipated that the thermal acceleration can be expanded to the other passive clearing methods and future research is needed.

## Conclusion

The slow speed is a common limitation for passive clearing methods. In this study, the elevated-temperature-induced acceleration of PACT was demonstrated. To screen the alternative temperature, we observed the tissue transparency and imaged the fluorescent neurons of *Thy1*-GFP-M mouse brain block at five temperature points, and found that the temperature range of 42–47 °C for PACT gave more excellent transparency and deeper imaging depth than 37 °C and comparable fluorescence preservation. The tissue size and cell morphology and fine structure keep almost the original state with minimal change after clearing. In addition, this method is also proved to be compatible with immunostaining for brain tissue blocks. The quantitative and systematic assessments of temperature based on PACT is supposed to provide alternative temperature range, which can simultaneously speed up clearing process and preserve fluorescence intensity. It may be expanded to other clearing methods based on long-time incubation.

## Methods

### Preparation of mouse brain blocks

Adult *Thy1*-GFP-M line and *Cx3Cr1*-GFP line mice (8–9 weeks old, Jackson Laboratory, USA) were used in this study. *Thy1*-GFP-M mice were employed to study the change of fluorescence intensity with time and different temperature, and *Cx3Cr1*-GFP were used to measure the imaging depth due to the dense distribution of microglia in different regions of the brain. Mice were anesthetized with a mixture of 2% α-chloralose and 10% urethane (8 mL/kg) through intraperitoneal injection, and perfused intracardially with 0.01 M phosphate buffered saline (PBS, Sigma) followed by 4% paraformaldehyde (PFA, Sigma-Aldrich) in PBS. The brains were excised and post-fixed overnight at 4 °C in 4% PFA. The mouse brains were rinsed for several times with PBS and then were sliced into 1-mm-thick coronal blocks with a vibratome (Leica VT 1000 s, Germany). All animal care and all experimental protocols were in accordance with the Experimental Animal Management Ordinance of Hubei Province, P. R. China and the guidelines from the Huazhong University of Science and Technology, and have been approved by the Institutional Animal Ethics Committee of Huazhong University of Science and Technology.

### PACT clearing protocol

The samples were cleared with PACT as described in the literature^32^,^33^. Before clearing, the brain sections were cut along the midline and incubated in A4P0 hydrogel monomer solution at 4 °C overnight. After infusion, the samples were degassed with nitrogen through the sample-hydrogel solution in the centrifuge tube for 5 to10 minutes and polymerized in 37 °C water-bath for 3 hours. Then the samples were rinsed with PBS, removed the excess hydrogel and placed into 50 ml conical tubes containing 8% SDS solution.

### Selection of clearing temperature

In this study, temperatures from 37 °C to 57 °C at the interval of 5 °C were chosen as the SDS clearing temperature. For recording the transparency along with time, the bright-field images were taken on the same 1-mm-thick GFP mouse brain block during SDS clearing at 12-hr, 24-hr, 36-hr, or 48-hr for different temperatures until cleared. To illustrate the increase of tissue transparency induced by sorbitol with refractive index matching, the 1-mm-thick brain sections were cleared in SDS solution for 6 hours, then collimated transmittance were measured before and after sorbitol incubation. The fluorescence images for each temperature were also taken on GFP mouse brain slices before and after clearing with SDS. To measure the imaging depth for different temperatures, the 1-mm-thick *Cx3Cr1*-GFP mouse brain blocks were cleared in SDS solution at 37 °C, 42 °C and 47 °C for 6 hours and 12 hours, respectively, and rinsed with PBST (0.1% Triton X-100 in PBS), then incubated in sorbitol solution (70% wt/vol) for 5 hours to achieve refractive index matching prior to imaging. The 3-mm-thick *Cx3Cr1*-GFP mouse brain blocks were also processed in SDS solution for 3 days with 37 °C, 42 °C and 47 °C, then rinsed with PBST, incubated in sorbitol solution for 5 hours before imaging.

### Immunostaining of PACT-processed mouse brain tissue

To demonstrate whether the temperature rise during PACT clearing influences the binding of antibody and antigen, the immunostaining was performed on 1-mm-thick *Thy1*-GFP-M brain section referring to the original PACT method^31^. Before staining, the 1-mm-thick slices were cleared with 8% SDS at 37 °C, 42 °C, and 47 °C, respectively. Then the previously cleared samples were immunostained for parvalbumin and nuclei stained with DAPI. After washed in PBST for 1 day and blocked in PBS/0.1% Triton X-100/6% goat serum for 1 day, the samples were transferred to primary antibody dilutions (anti-parvalbumin antibody, Abcam, ab11427, 1:400) for 1 day followed by washing with PBST for several times, then to secondary antibody dilutions (Alexa Fluor 647 goat anti-rabbit IgG, Jackson Immunoresearch, 111-607-003, 1:400) for 1 day at 37 °C. Then the samples were nuclei stained with DAPI at room temperature for 12 hours. The samples were finally washed in PBST for several times before further incubating in 70% (wt/vol) sorbitol solution for 1 or 5 hours. For GFP immunostaining, the cleared brain slice was incubated in primary antibody dilutions (anti-GFP antibody, Millipore, AB3080, 1:200) for 2 days and secondary antibody dilutions (Alexa Fluor 633 goat anti-rabbit IgG, Invitrogen, A-21070, 1:200) for 2 days.

### Fluorescence microscopy

The clarified brain slices were mounted with two cover glasses and imaged with the inverted confocal fluorescence microscopy (LSM710, Zeiss, Germany) equipped with the Fluar 10×/0.5 objective (dry, W.D. 2.0 mm) and Plan-Apochromat 20×/0.8 objective (dry, W.D. 0.55 mm) or upright confocal fluorescence microscopy (A1RMP, Nikon, Japan) equipped with the 16×/0.8 water-immersion objective (W.D. 3.0 mm).

### Measurement of collimated light transmittance

Light transmittance of the 1-mm-thick mouse brain sections were measured with a commercially available spectrophotometer (Lambda 950, PerkinElmer, USA). Due to the small size of half brain slices, a customized 3 mm × 3 mm slit was designed to obtain the collimated transmittance spectra (400–800 nm). The light transmittance of samples normalized to the blank value, which was 100%, defined as the relative collimated transmittance.

### Imaging data processing

The obtained images were analyzed with ImageJ software and Imaris software. For fluorescence quantification, the freehand-selection tool was used to select the soma area of a neuron, and the histogram tool was used to measure the mean fluorescence intensity and the area, whose multiplication values were served as the total fluorescence intensity of the neurons. The fluorescence intensity of a neuron is supposed to be ‘A’ before clearing and ‘B’ after clearing. The fluorescence change of a neuron during clearing is quantified as ‘B/A’. For each group, mean value of fluorescence change of twenty-five neurons was calculated. For sample expansion rate, based on the bright-field images, the size of the slices was outlined using ImageJ software. The linear expansion value was determined by normalizing the area of cleared slices with the area of uncleared ones and calculating the square root (n = 4). For imaging depth evaluation, the decay of image contrast with depth was used to quantify the value after clearing in different temperatures. The image contrast was calculated as the following Eq. (1) 4,45.


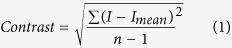


where I is the grayscale value for each pixel, I_mean_ indicates the average intensity of each frame, and n is the total pixel count. Data were analyzed and graphs constructed using Matlab, Microsoft Excel or Visio. Statistical analysis was performed using one-way ANOVA followed by Tukey’s post hoc test or two-way ANOVA with mixed design.

## Additional Information

**How to cite this article**: Yu, T. *et al*. Elevated-temperature-induced acceleration of PACT clearing process of mouse brain tissue. *Sci. Rep.* **7**, 38848; doi: 10.1038/srep38848 (2017).

**Publisher's note:** Springer Nature remains neutral with regard to jurisdictional claims in published maps and institutional affiliations.

## Supplementary Material

Supplementary Information
