# Supplementary material for: Elevated-temperature-induced acceleration of PACT clearing process of mouse brain tissue
Source: Sci Rep. 2017 Jan 31;7:38848. doi: 10.1038/srep38848 (PMC5282525; doi:10.1038/srep38848)
Supplement: Supplementary Information [file srep38848-s1.pdf]

Elevated-temperature-induced acceleration of PACT clearing process of mouse brain tissue

Tingting Yu[1,2,+], Yisong Qi[1,2,+],Jingtan Zhu[1,2], Jianyi Xu[1,2], Hui Gong[1,2], Qingming Luo[1,2], Dan Zhu[1,2,*]

1. Britton Chance Center for Biomedical Photonics, Wuhan National Laboratory for Optoelectronics, Huazhong University of Science and Technology, 1037 Luoyu Road, Wuhan, Hubei 430074, China

2. Department of Biomedical Engineering, Key Laboratory for Biomedical Photonics, Ministry of Education, Huazhong University of Science and Technology, 1037 Luoyu Road, Wuhan, Hubei 430074, China

+these authors contributed equally to this work

*Corresponding author:

Dan Zhu, Ph.D, Professor

Email: dawnzh@mail.hust.edu.cn

Tel: +86-02787792033-302, Fax: +86-02787792034

Supplementary Figure 1

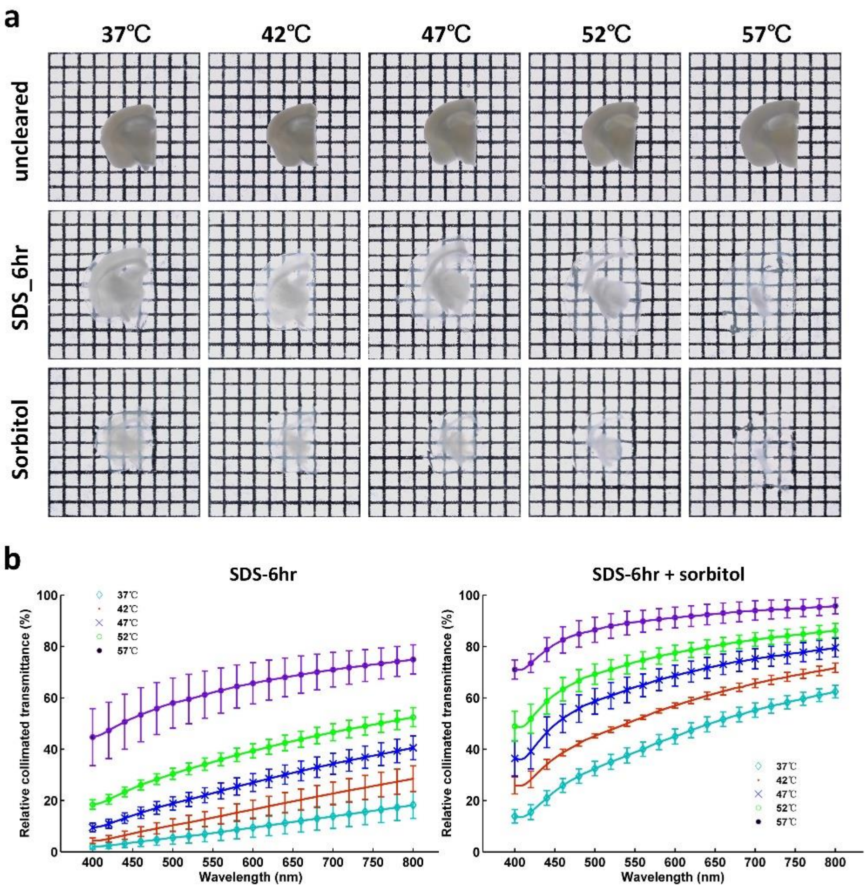

Supplementary Figure 1. (a) Bright-field images of 1-mm-thick brain sections cleared in SDS for 6 hours and further incubated in sorbitol solution. “uncleared” indicates the sample with only hydrogel-embedded and placed in PBS. “SDS-6hr” indicates 6-hours SDS clearing. “Sorbitol” indicates further incubation in sorbitol solution after 6-hours SDS clearing. **(b)** Relative collimated transmittance of brain blocks after 6-hours SDS clearing and further sorbitol incubation. “SDS-6hr” indicates the sample with 6-hours SDS clearing, “SDS-6hr + sorbitol” indicates the sample with further sorbitol incubation after 6-hours SDS clearing. Error bars denote standard deviations.

Supplementary Figure 2

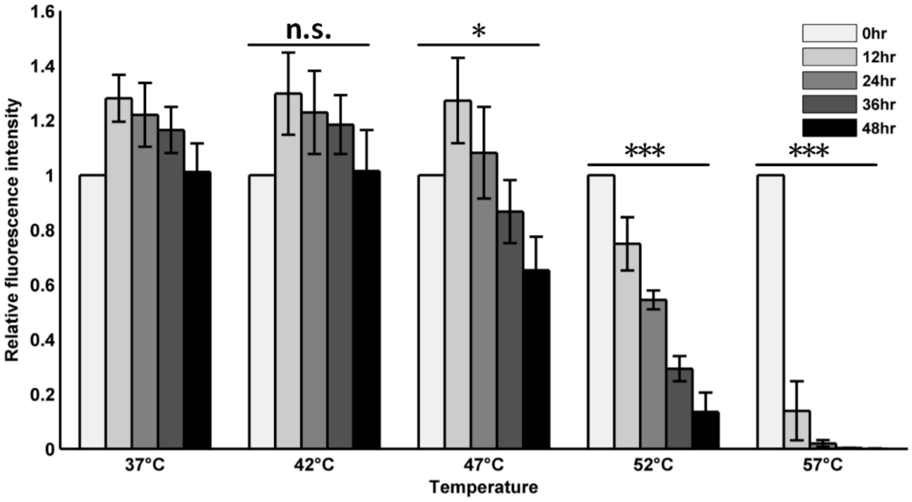

Supplementary Figure 2. Quantitative calculation of relative fluorescence intensity. Error bars denote standard errors. Statistic method: two-way ANOVA with mixed design. n.s., not significant; * $P<0.05$; *** $P<0.001$. The statistical analyses show that the difference of fluorescence change within 48 hours between 42 °C and 37 °C is not significant, while for 47 °C, the fluorescence change is significantly different from 37 °C. 52 °C and 57 °C present highly significant difference with 37 °C.

Supplementary Figure 3

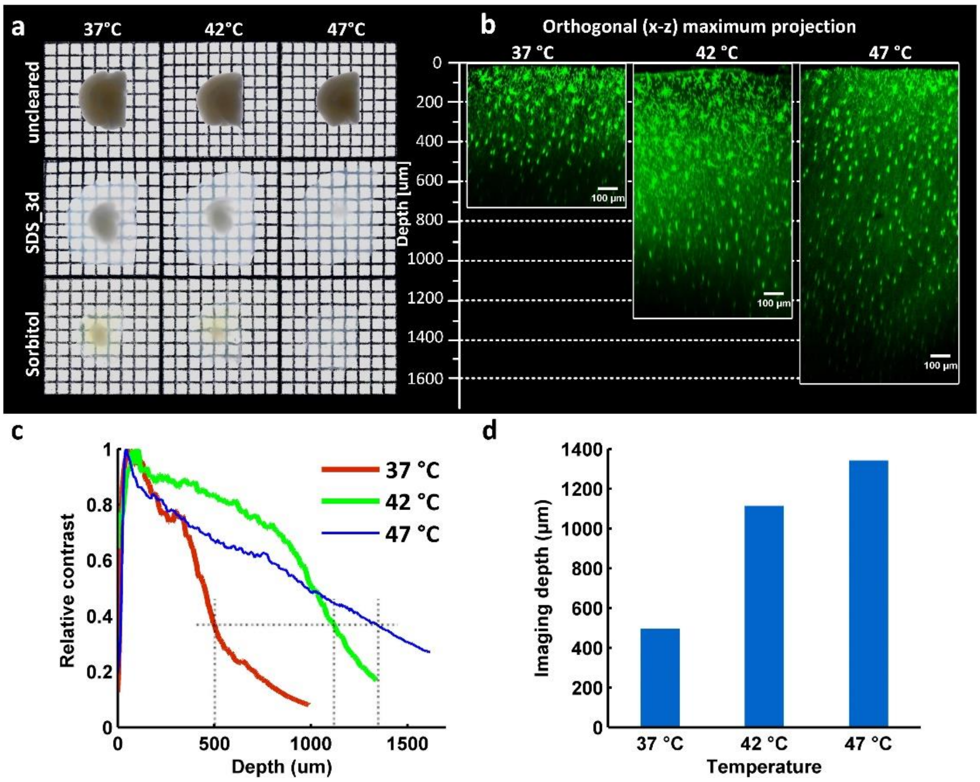

Supplementary Figure 3. To further investigate the effect of temperature on imaging depth of larger samples, 3-mm-thick brain blocks were cleared with SDS for 3 days followed by sorbitol incubation, then imaged with upright confocal fluorescence microscopy (A1RMP, Nikon, Japan) equipped with the 16×/0.8 water-immersion objective (W.D. 3.0 mm). **(a)** Transparency of the brain blocks after 3-days SDS clearing with 37 °C, 42 °C and 47 °C and sorbitol immersion. The opaque area of the brain blocks decreases with increasing temperature. **(b)** The 20-μm maximum projections of orthogonal (x-z) slices, taken from z-stack transverse images of the center region of cleared 3-mm-thick brain blocks. **(c-d)** Quantification of imaging depth. The imaging depth is 492 μm, 1120 μm and 1332 μm for three temperatures, respectively. For 42 °C and 47 °C, the value increases to 2.28 and 2.71 folds, respectively.

Supplementary Figure 4

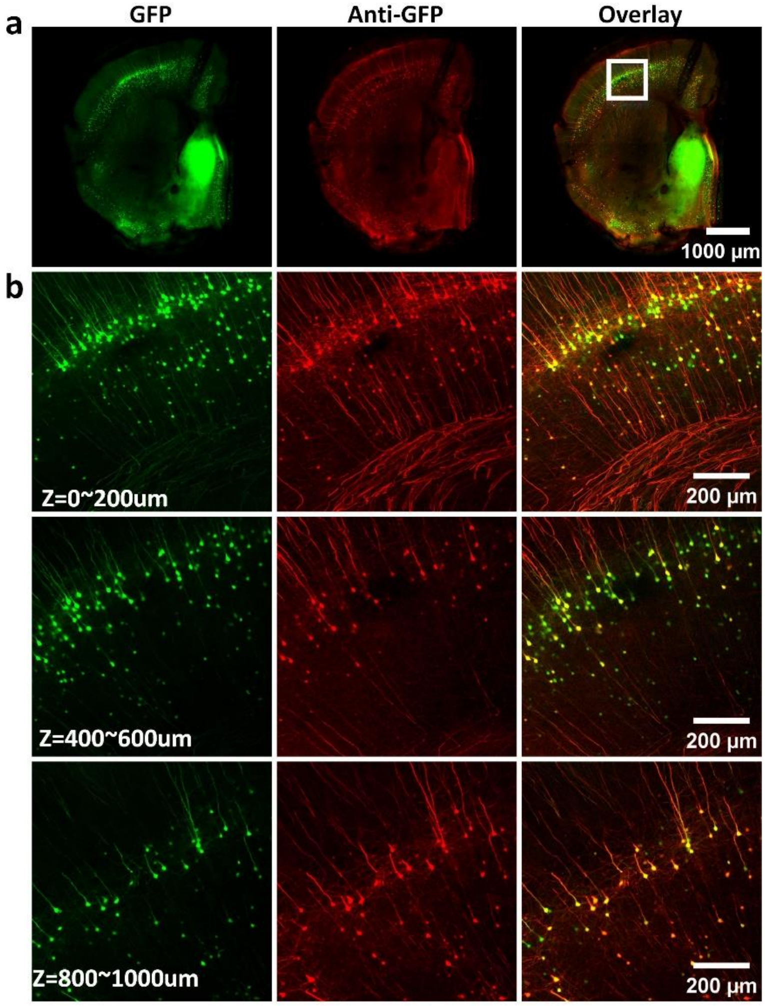

Supplementary Figure 4. Immunostaining for *Thy1*-GFP-M of 1-mm-thick mouse block after PACT clearing at 47 °C. **(a)** Single plane images of coronal block of GFP mouse brain immunostained for GFP. Scale bar, 1000 μm. **(b)** Maximum projections of the boxed region in the cortex in (a) at different depths (thickness: 200 μm). Scale bar, 200 μm. Left, endogenous GFP (green); middle, anti-GFP (red); right, overlay. The signals on the coronal surface and middle of the brain block overlap well, which indicates that moderately rising temperature (e.g. 47 °C) for PACT works well with subsequent immunostaining.

Supplementary Figure 5

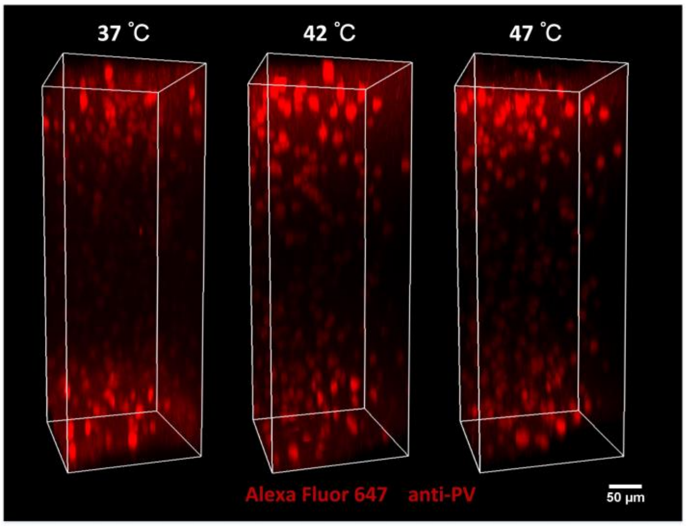

Supplementary Figure 5. Immunostaining of 1-mm-thick brain slices. Cleared brain slices with PACT in different temperatures were immunostained for parvalbumin (Alexa Fluor 647 anti-PV), then incubated in sorbitol solution for 1 h and mounted for imaging.

Supplementary Figure 6

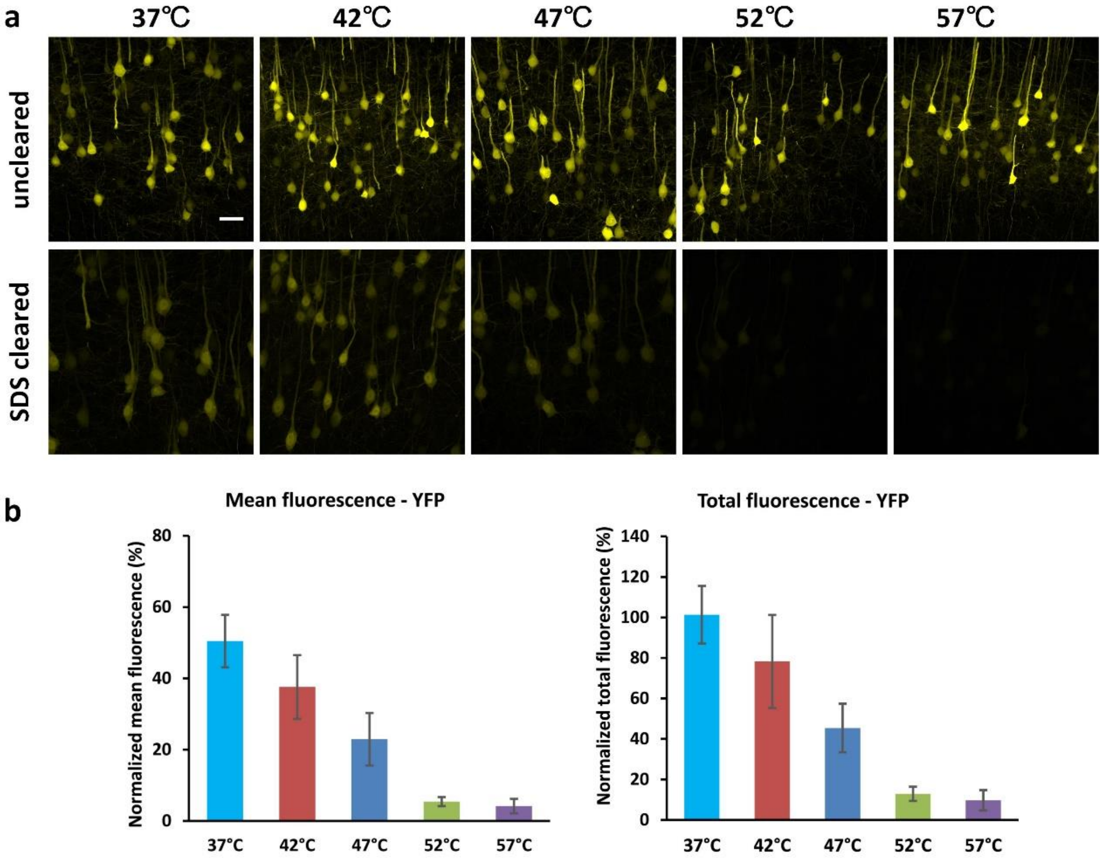

Supplementary Figure 6. YFP Fluorescence intensity of neurons before and after SDS clearing in gradient temperature. **(a)** Each image is the maximum projection of image stacks acquired with confocal microscope (10×/0.5 objective). Scale bar, 50 µm. **(b)** Quantitative calculation of relative fluorescence intensity. Error bars denote standard deviations.
